# Intraoperative indocyanine green fluorescence cholangiography can rule out biliary atresia: A preliminary report

**DOI:** 10.3389/fped.2022.1005879

**Published:** 2022-11-04

**Authors:** Chiyoe Shirota, Akinari Hinoki, Takao Togawa, Shogo Ito, Wataru Sumida, Satoshi Makita, Hizuru Amano, Aitaro Takimoto, Shunya Takada, Masamune Okamoto, Yoichi Nakagawa, Daiki Kato, Hiroo Uchida

**Affiliations:** ^1^ Department of Pediatric Surgery, Nagoya University Graduate School of Medicine, Nagoya, Japan; ^2^ Department of Rare/Intractable Cancer Analysis Research, Nagoya University Graduate School of Medicine, Nagoya, Japan; ^3^ Department of Pediatrics and Neonatology, Nagoya City University Graduate School of Medical Sciences, Nagoya, Japan

**Keywords:** biliary atresia, ICG fluorescence cholangiography, diagnosis, neonate, cholestasis

## Abstract

**Background:**

The prognosis of BA is known to be poor if definitive surgery is performed too late. Therefore, excluding BA as a diagnosis at an early stage is crucial. Conventional cholangiography requiring cannulation through the gallbladder may be unnecessarily invasive for patients, especially when ruling out BA. Therefore, a less invasive alternative such as indocyanine green (ICG) cholangiography, which does not require cannulation, should be established. In this study, we focused on excluding BA and confirmed the usefulness of intravenous ICG fluorescence cholangiography. To the best of our knowledge, this is the first preliminary study to report the use of intravenous ICG cholangiography for BA exclusion.

**Methods:**

The study participants were patients who underwent liver biopsy and intraoperative cholangiography after they were suspected to have BA, between 2013 and 2022. ICG fluorescence cholangiography was performed on all patients who provided informed consent.

**Results:**

During the study period, 88 patients underwent a laparoscopic liver biopsy and cholangiography. Among them, 65 (74%) were diagnosed with BA and underwent a subsequent laparoscopic Kasai portoenterostomy. BA was ruled out intraoperatively in 23 patients. Of the 23 patients in whom BA was ruled out, 14 underwent ICG cholangiography, 11 had gallbladder (GB) fluorescence, and 9 had both GB and common bile duct (CBD) fluorescence. Conventional cholangiography was very difficult in 2 of 23 cases: in 1 case, cannulation of the atrophic gallbladder was impossible, and cholecystectomy was indicated after multiple attempts; in 1 case, upstream cholangiography was not possible. In both cases, ICG fluorescence cholangiography successfully imaged the CBD and the GB.

**Conclusions:**

In conclusion, intravenous ICG fluorescence cholangiography might be a useful and less invasive diagnostic procedure that can rule out BA in infants.

## Introduction

The prognosis of BA is known to be worse if definitive surgery is performed too late (1, 2). Therefore, early diagnosis of BA is critical in infants with cholestasis (1, 3–5).

A definitive diagnosis of BA can be established by histopathological examination of liver biopsy samples, and intraoperative cholangiography. Therefore, some articles recommend performing surgery for diagnosis at an early stage without additional preoperative testing such as percutaneous liver biopsy or magnetic resonance cholangiopancreatography (MRCP) (6). Our strategy is to perform intraoperative histopathological examination of liver biopsy samples and intraoperative cholangiography with a laparoscope, and proceed directly to radical surgery for BA if diagnosed.

MRCP, computed tomography (CT), and cholescintigraphy are sometimes used as less-invasive diagnostic tools for BA. While cholescintigraphy is a dynamic test that confirms the excretion of bile from the liver, MRCP and CT can only detect bile duct morphology. Therefore, MRCP and CT can neither confirm bile excretion nor diagnose BA. Cholescintigraphy is a less invasive diagnostic tool that allows the uptake of intravenously administered technetium by the liver and its excretion in the bile (7). However, in cases of severe biliary congestion, technetium excretion may not be seen even in the absence of BA and is not sensitive enough to reliably diagnose BA (8).

Conventional cholangiography requires cannulation of the gallbladder. However, an atrophic gallbladder can sometimes preclude cannulation, retrograde cholangiography sometimes fails to demonstrate images of the upstream bile ducts (9). Once BA is diagnosed, a subsequent cholecystectomy is performed as a radical operation; therefore, invasive cannulation should not be a problem. However, when BA is ruled out, a subsequent cholecystectomy is not warranted, and conventional cholangiography requiring cannulation through the gallbladder may be unnecessarily invasive for patients; therefore, a less invasive alternative should be established.

In contrast, in intravenous ICG fluorescence cholangiography, ICG is 100% taken up entirely by hepatocytes after intravenous injection, and subsequently excreted in the bile (10, 11). Thus, we hypothesize that if the fluorescence of the common bile duct (CBD) after intravenous injection of ICG can be confirmed, BA can be excluded or diagnosed in a manner similar to biliary scintigraphy.

In this study, we focused on excluding BA and confirming the utility of intravenous indocyanine green (ICG) fluorescence cholangiography. To the best of our knowledge, this is the first study to report the use of intravenous ICG cholangiography for the exclusion of BA. Here, we report favorable outcomes of ICG fluorescence cholangiography compared with conventional cholangiography.

## Materials and methods

### Patients

This study was performed after obtaining approval from our institutional review board and clinical investigation review committee (Ethics Review Board at Nagoya University Graduate School of Medicine approval No. 2018-0472) and followed the Declaration of Helsinki. Written informed consent for publication of their details was obtained from the guardian. The study participants were pediatric patients who underwent liver biopsy and intraoperative cholangiography after suspected BA between October 1, 2013, and February 31, 2022. ICG fluorescence cholangiography was performed in patients whose parents or guardians provided informed consent. Our strategy is to perform intraoperative histopathological examination of liver biopsy samples and intraoperative cholangiography with a laparoscope, and proceed directly to radical surgery for BA if diagnosed. Because it is difficult to make a definitive diagnosis of BA by intraoperative histopathological examination alone, the definitive diagnosis of BA is made by intraoperative cholangiography. If the diagnosis of BA is made by intraoperative cholangiography, the results of intraoperative histopathological examination should confirm findings that support the diagnosis of BA, such as pseudobiliary duct proliferation. In our country, ICG fluorescence cholangiography is considered an off-label method; therefore, ICG fluorescence cholangiography was only performed on a trial basis in addition to conventional cholangiography in patients who consented to its use during the current study period.

### Surgical procedure

**Figure 1:**
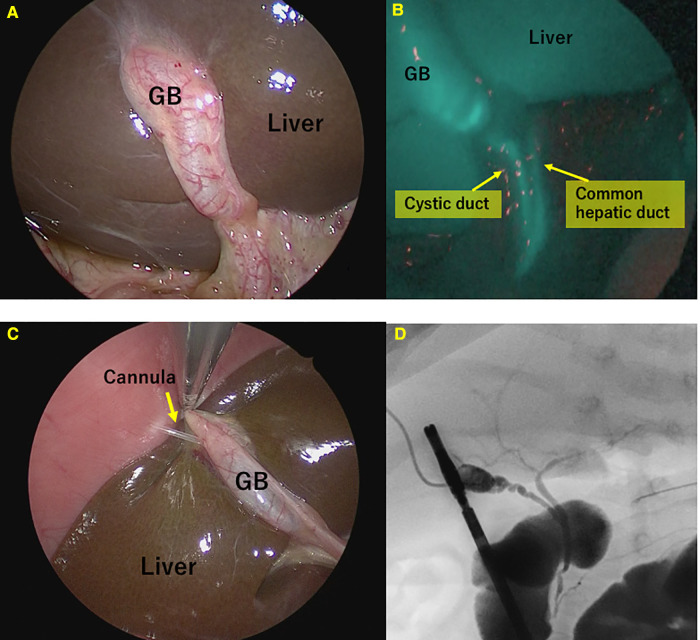
Fluorescence and conventional cholangiography for non-biliary atresia (BA) patients. (**A**) Normal view. (**B**) Fluorescence cholangiography. **(C)** Cannulation of the gallbladder. (**D**) Conventional cholangiography.

Single-port surgery, conducted only through the umbilical opening, was performed until the BA was definitively diagnosed. A multichannel port was inserted through the umbilical wound, one 5-mm camera port, and two 3-mm ports for the surgeon's right and left forceps. First, we confirmed the gross liver findings (Figure 1A). An intraoperative liver biopsy was first performed for intraoperative pathological diagnosis, and after hemostasis of the liver tissue, ICG fluorescence cholangiography was performed to observe the gallbladder and bile ducts(Figure 1B). The camera was placed in close proximity to the extrahepatic bile duct or slightly further away to observe the entire image, changing the distance several times so that the gallbladder to the duodenum was in the field of view at 1x. Although ICG fluorescence cholangiography showed that the bile ducts could be observed and BA could be ruled out, conventional cholangiography was still performed. The cannula was inserted *via* a gallbladder puncture either percutaneously or after being pulled out in the abdominal cavity(Figure 1C). Subsequently, the contrast agent was injected under radiographic guidance to confirm bile flow (Figure 1D).

### ICG fluorescence cholangiography

Since the time of ICG excretion from the liver depends on the severity of biliary stasis, we considered that the optimal period for ICG injection may vary greatly from patient to patient. We tested three options for timing of administration (24 h before surgery, 1 h, and both 24 h and 1 h) based on previous reports (12–16).

To determine the optimal time point of ICG injection, ICG was injected preoperatively at the dose of 0.05 mg/kg/time point at 24 h, 1 h, or both. ICG fluorescence cholangiography was performed using fluorescence imaging equipment for laparoscopy (IMAGE1 S™ NIR/ICG, Karl Storz SE & Co, KG, Tuttlingen, Germany) after liver biopsy. As an exploratory endpoint, patients who underwent ICG fluorescence cholangiography also underwent conventional cholangiography with gallbladder cannulation.

Fluorescence of the liver was considered “faint” if it was visible but faint and not clearly demarcated from the surrounding area. Fluorescent cholangiography was defined as “fluorescent +” if the fluorescence of GB and CBD was equivalent to that of the liver and the boundaries were clear, and “fluorescent −” if not.

## Results

During the study period, 88 patients underwent a laparoscopic liver biopsy and cholangiography. Among them, 65 (74%) were diagnosed with BA and underwent subsequent laparoscopic Kasai portoenterostomy directly. BA was ruled out intraoperatively in the remaining 23 patients (19 boys and four girls). The 23 patients in whom BA was ruled out had a median age of 61 days (range, 34–201 days), the median duration of surgery was 81 min (range, 58–90 min), blood loss during surgery was 1 ml (range, 0–39 ml), and direct bilirubin level was 5.4 mg/dl (1.2–12.4 mg/dl).

**Figure 2:**
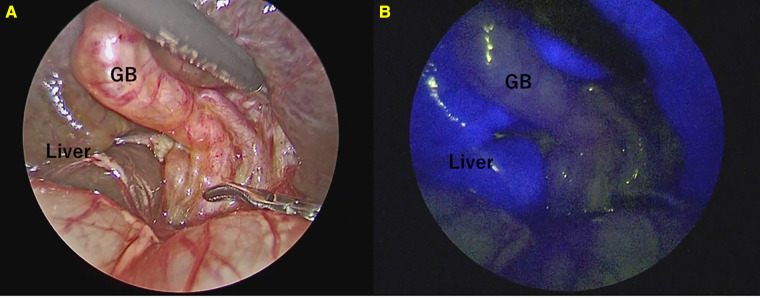
Fluorescence cholangiography in patients with biliary atresia. Only the liver appeared to fluoresce strongly. (**A**) Normal view. (**B**) Fluorescence cholangiography.

ICG fluorescence cholangiography was performed in 43 of 65 (type I: three cases; type III: 62 cases) patients diagnosed with BA; only the liver and not the gallbladder or common bile duct were imaged in all cases (Figure 2). The conventional cholangiography also did not detect visualization of Extrahepatic bile duct. In other words, the specificity of ICG fluorescence cholangiography for the diagnosis of BA exclusion was 100% (43/43).

Among the 23 patients in whom BA was ruled out, ICG fluorescence cholangiography was performed in 14. ICG was preoperatively injected at a dose of 0.05 mg/kg/time point at 24 h, 1 h, or 24 h and 1 h prior to surgery in one, four, and nine cases, respectively. Fluorescence of both GB and CBD was observed in 9 of the 14 cases, and only the GB was observed in 2 cases. In the remaining 3 cases, neither the GB nor the CBD was fluorescent (Table 1). The sensitivity of ICG fluorescence cholangiography for BA exclusion diagnosis was 64.3% (9/14). Cholescintigraphy was performed in 6 of the 14 cases in which ICG fluorescent cholangiography was performed, and only 1 of the 6 cases showed extrahepatic excretion (Table 1). One case with extrahepatic excretion of bile on cholescintigraphy was not considered to be BA, but the direct bilirubin remained elevated and the cause could not be determined, hence, laparoscopic liver biopsy and intraoperative cholangiography were planned.

**Table 1:** The 14 patients who underwent ICG cholangiography from among the 23 patients for whom BA was ruled out.

Sex	Age at the surgery (days)	BW (Kg)	Diagnosis	ICG injection (0.05 mg/Kg)		ICG Fluorescent cholangiography			Convensional cholangio-graphy+ fluorescent	Choles-cintigraphy	D-bil (mg/dl)
				24 h pre-operation	1 h pre-operation	Liver	GB − failure	CBD + success		− no fluorescent	
F	64	3.8	Citrin deficiency	◯	X	faint	−	−	+	−	2.5
F	76	4.1	Citrin deficiency	◯	◯	faint	−	−	+		5.8
F	52	2.8	Not definitive	X	◯	+	−	−	+	−	6.2
M	59	4.7	CMV infection	◯	◯	+	+	−	+		8.7
M	66	4.5	CMV infection	◯	◯	+	+	−	+		10
M	201	2.4	Bile acid synthesis defects	X	◯	+	+	+	−	−	1.6
M	75	5.8	CMV infection	X	◯	+	+	+	+		1.8
M	34	3.8	Not definitive	X	◯	+	+	+	+	−	3.8
M	75	3.8	Alagille syndrome	◯	◯	+	+	+	−	−	4.4
F	43	3.7	Alagille syndrome	◯	◯	+	+	+	+		5.8
M	61	3.9	Not definitive	◯	◯	+	+	+	+		1.2
M	77	4.4	Not definitive	◯	◯	+	+	+	+	+	7.7
M	60	4.2	Not definitive	◯	◯	+	+	+	+		9.2
M	44	4.4	Not definitive	◯	◯	+	+	+	+		5.4

BAD, bile acid synthesis defects.

**Figure 3:**
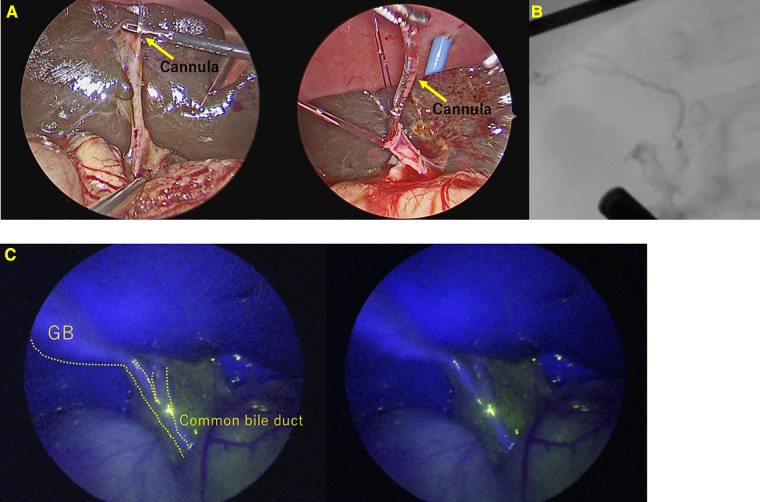
A case of Alagille syndrome requiring cholecystectomy after contrast due to difficulty in contrast from an atrophic gallbladder. (**A**) Cannulation into the gallbladder was difficult and took many attempts. (**B**) Conventional cholangiography. (**C**) ICG Fluorescence cholangiography.

Conventional cholangiography was performed in all 23 cases that were not BA. Conventional cholangiography was extremely difficult in 2 of the 23 cases(8.7%) in whom BA was ruled out. In one case, the common hepatic duct could not be visualized. In another case, cannulation from the gallbladder was difficult to perform, although we managed to obtain contrast, ultimately requiring cholecystectomy. (Figure 3) In both cases where conventional cholangiography was difficult, both CBD and GB fluoresced clearly on ICG fluorescence cholangiography.

Eleven of the 23 patients for whom BA was ruled out showed a trend toward postoperative recovery of jaundice and eventually recovered from jaundice completely during the observation period. The remaining 12 patients, who did not recover from jaundice, were examined for potential genetic disorders. Genetic examination identified 11 cases of genetic disorders: Dubin–Johnson syndrome in 2, citrin deficiency in 2, Alagille syndrome in 2, trisomy 21 in 2, bile acid synthesis defects in 1, and two genetic variants of unknown diagnostic significance (e.g., *ATP11C* and *ABCB11*) in 2 patients (Table 2).

**Table 2:** The 23 patients for whom BA was ruled out intraoperatively.

Case	Sex	Age at the surgery (days)	BW (Kg)	DB (mg/dl)	Operative time (min)	Blood loss (ml)	Diagnosis	Genetic disorders
1	M	48	4.4	6.1	79	1	Dubin-Johnson syndrome	ABCC2
2	M	64	4.6	6.7	58	1	Dubin-Johnson syndrome	ABCC2
3	F	64	3.8	2.5	87	5	Citrin deficiency	SLC25A13
4	F	76	4.1	5.8	65	1	Citrin deficiency	SLC25A13
5	M	75	3.8	4.4	198	4	Alagille syndrome	NOTCH2
6	F	43	3.7	5.8	74	1	Alagille syndrome	JAG1
7	M	201	2.4	1.6	84	1	BAD	CYP7B1
8	M	54	4.3	7	48	0	Not definitive	ATP11C
9	M	64	4.3	4.3	76	1	Not definitive	ABCB11
10	M	60	3.1	12.4	55	1	Trisomy 21	Trisomy 21
11	M	61	3.7	2.4	147	1	Trisomy 21	Trisomy 21
12	M	75	5.8	1.8	58	0	CMV infection	NO
13	M	59	4.7	8.7	68	0	CMV infection	NI
14	M	66	4.5	10	75	1	CMV infection	NI
15	M	98	5.8	2.6	90	39	Not definitive	NI
16	M	85	4.8	1.5	66	1	Not definitive	NI
17	M	38	3.7	2.6	50	1	Not definitive	NI
18	F	52	2.8	6.2	79	1	Not definitive	NI
19	M	61	3.9	1.2	121	1	Not definitive	NI
20	M	34	3.8	3.8	102	5	Not definitive	NI
21	M	77	4.4	7.7	110	1	Not definitive	NI
22	M	60	4.2	9.2	91	1	Not definitive	NI
23	M	44	4.4	5.4	74	1	Not definitive	NI

DB, direct bilirubin; BAD, bile acid synthesis defects; NO, no genetic disorders; NI, not inspected.

## Discussion

ICG fluorescence cholangiography has been widely applied as a bile duct imaging technique in surgeries for various hepatobiliary diseases, including cholelithiasis, cholangiocarcinoma, liver tumors, and pancreatic tumors in adults (9, 17,18). This technique is performed by injecting ICG either intravenously or through the gallbladder or the bile duct. Using near-infrared imaging equipment, ICG can be observed in tissues up to 10 mm thick (10).

To perform Intraoperative Indocyanine Green Fluorescence Cholangiography for ruling out BA, ICG must be administered intravenously. After intravenous injection, ICG is 100% taken up by hepatocytes, and excreted into the bile (10). In ICG fluorescence cholangiography, fluorescence of GB after intravenous ICG injection indicates bile excretion from the liver, so BA other than type I can be ruled out. In other words, fluorescence of GB indicates that the bile ducts upstream of GB are not obstructed. If CBD fluoresces, all types of BA can be ruled out.

In conventional cholangiography, a contrast agent is injected through the gallbladder, so BA cannot be ruled out unless both upstream and downstream contrast can be obtained. Upstream contrast is often particularly difficult. If the GB fluoresces in ICG fluorescent cholangiography, BA can be ruled out by simply confirming downstream contrast.

**Figure 4:**
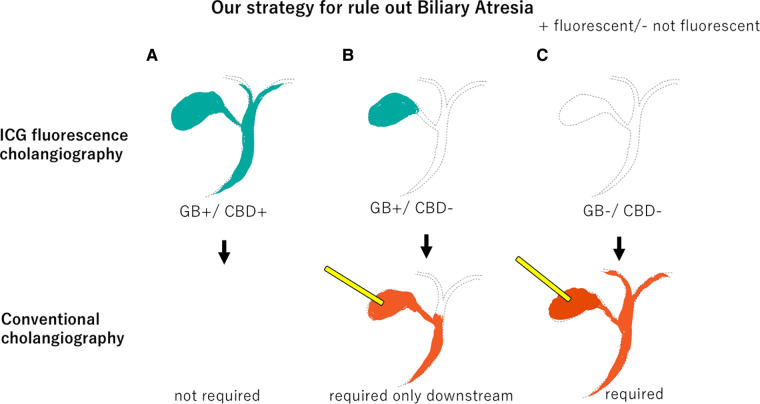
Our strategy to rule out Biliary Atresia. (**A**) If both the gallbladder and common bile duct fluoresce on ICG fluorescence cholangiography, conventional cholangiography is unnecessary. (**B**) If only the gallbladder fluoresces, only downstream should be confirmed by conventional cholangiography. (**C**) If neither the gallbladder nor the common bile duct fluoresces, both upstream and downstream should be confirmed by conventional cholangiography. GB, gallbladder.

The prognosis of BA is known to be worse if definitive surgery is performed too late (1, 2). However, a definitive diagnosis is difficult and often established based on intraoperative cholangiography and liver biopsy (3). Furthermore, conventional cholangiography requiring contrast injection through the gallbladder also presents technical challenges and imaging uncertainties (11, 19). Therefore, it is anticipated that intraoperative cholangiography cannot be easily undertaken, even if BA is suspected. ICG fluorescence cholangiography can be easily performed with transvenous ICG injection and does not require cannulation through the atrophic gallbladder or retrograde cholangiography. Intravenous ICG fluorescent cholangiography is a minimally invasive and useful diagnostic method to exclude BA in infants with cholestasis, and we believe that conventional cholangiography should be performed only when this diagnostic method fails to make a diagnosis. If the GB is fluorescent, upstream contrast is not mandatory, even for conventional cholangiography (Figure 4). ICG fluorescence cholangiography makes performing intraoperative cholangiography easier than when conventional cholangiography was the only option, and it is expected to lead to earlier intraoperative cholangiography and curative surgery.

In this study, ICG fluorescent cholangiography was successful in visualizing bile excretion from the liver, as the GB fluoresced in 11 of 14 cases. In contrast, neither GB nor CBD showed fluorescence in the remaining three cases. In three cases, neither GB nor CBD fluoresced; two of the three had citrin deficiency (NICCD). In these two cases, liver fluorescence was faint. This suggests that in patients with metabolic diseases such as NICCD, the uptake of ICG into cells may be weak. However, this consideration cannot be strongly supported because the fluorescence intensity is subjective and cannot be quantified.

Few studies have reported the outcomes of ICG fluorescence cholangiography in pediatric patients. Therefore, the optimal dose level or timepoint(s) for ICG injection has not been established (20). Additionally, based on our experience with cholescintigraphy, ICG elimination time differs depending on the severity of cholestasis; therefore, the optimal time for ICG injection could vary widely among patients. Based on reports on adult patients, we selected a dose level of 0.05 mg/kg (21). The dose level in our trial was lower than that reported in previous studies for ICG navigation surgery of BA; however, it was sufficient to observe bile flow (13, 15, 16). The timing of administration was tested using three options (24 h, 1 h, or both 24 and 1 h preoperatively).

The number of cases in which BA was suspected, and the diagnosis sought to be excluded, was extremely small, and the small number of cases in the present study is a limitation. Although the current assessment was preliminary with a limited number of patients, as patients who were injected with ICG twice (24 and 1 h preoperatively) exhibited adequate fluorescence in both the liver and gallbladder, the regimen of ICG injections twice may reduce the risk of failure of adequate cholangiography imaging. Various reports in adult patients without cholestasis indicate that the optimal time point of ICG injection is 1 h before observation; however, the elimination rate could be decreased depending on the severity of cholestasis (21). Thus, we plan to select two injection time points, that is, 24 and 1 h preoperatively, as a standard protocol.

In conclusion, ICG fluorescence cholangiography was validated as a minimally invasive and reliable method to rule out BA. The specificity was 100%, but the sensitivity was rather low at 64.3%, making it difficult to exclude BA by itself. However, this procedure was considered to be worthwhile, because it eliminated the need for conventional cholangiography in more than 60% of cases. We hope to continue validation and improve sensitivity by developing better protocols. If the sensitivity of ICG fluorescence cholangiography is improved by the revised protocol, we believe that early diagnosis by minimally invasive ICG fluorescence cholangiography will enable early radical surgery, as this is the most important step in early radical surgery of BA, rather than delaying surgery to make a diagnosis with numerous examinations.

## Data Availability

The raw data supporting the conclusions of this article will be made available by the authors, without undue reservation.
